# Antioxidant insights: investigating the protective role of oxidative balance in inflammatory bowel disease

**DOI:** 10.3389/fendo.2024.1386142

**Published:** 2024-05-31

**Authors:** Fan Li, Yu Chang, Zhaodi Wang, Zhi Wang, Qi Zhao, Xiaoping Han, Zifeng Xu, Chanjiao Yu, Yue Liu, Shiyu Chang, Hongyan Li, Sileng Hu, Yuqin Li, Tongyu Tang

**Affiliations:** ^1^ Department of Gastroenterology, The First Hospital of Jilin University, Changchun, China; ^2^ Norman Bethune Health Science Center, Jilin University, Changchun, China

**Keywords:** inflammatory bowel disease, oxidative balance score, UK biobank, Crohn disease, ulcerative colitis

## Abstract

**Background:**

Limited studies have investigated the relationship between systemic oxidative stress and inflammatory bowel disease (IBD). The purpose of this study was to explore the relationship between oxidative balance score (OBS) and IBD.

**Methods:**

We included 175,808 participants from the UK Biobank database from 2006 to 2010. OBS scores were calculated based on 22 lifestyle and dietary factors. Multiple variable Cox proportional regression models, as well as gender stratification and subgroup analysis, were utilized to investigate the relationship between OBS and IBD.

**Results:**

There is a significant negative correlation between OBS and the occurrence of IBD, ulcerative colitis (UC), and Crohn’s disease (CD). Additionally, OBS is significantly negatively correlated with intestinal obstruction in CD patients. Gender stratified analysis suggest a significant correlation between OBS and CD in female patients, particularly pronounced in those under 60 years old. Sensitivity analysis indicates a significant negative correlation between lifestyle-related OBS and diet-related OBS with the occurrence of CD in females, diet-related OBS is negatively correlated with CD in males.

**Conclusion:**

OBS showed a significant negative correlation with IBD, especially in female CD patients. This study underscores the importance of antioxidant diet and lifestyle, which may provide a greater advantage for female CD patients.

## 1 Introduction

Inflammatory bowel disease (IBD) is a group of lifelong autoimmune diseases, including Crohn’s disease (CD) and ulcerative colitis (UC). By 2019, there were 4.9 million cases of IBD worldwide (1). IBD affects the most productive period of a person’s adulthood, not only imposing a significant economic burden, but also impacting the psychological and social well-being of patients (2).

In recent years, researchers in the field of IBD have shown increasing interest in the role of oxidative stress. Early studies have demonstrated that patients with active IBD exhibit significantly elevated levels of oxidative stress, which is closely associated with the severity of intestinal inflammation. Oxidative stress not only increases the production of inflammatory mediators but also damages the intestinal barrier function. This impairment facilitates the penetration of pathogens and toxins, exacerbating the pathological process of IBD (3, 4). Numerous studies have demonstrated that elevated levels of reactive oxygen species (ROS) or decreased levels of antioxidants can promote the development of IBD (5, 6). In IBD patients, the intestinal mucosa is typically infiltrated by a large number of neutrophils, macrophages, and lymphocytes, and these inflammatory cells produce ROS and reactive nitrogen species (RONS) that can attack the cell membrane lipids, proteins, and DNA of intestinal mucosal cells, thereby exacerbating the progression of IBD (7–9).

RONS are produced as a result of self-inflammatory responses, as well as from exogenous sources such as diet, lifestyle, and environmental toxins (10). Due to the complexity of the human body and the multiple mechanisms involved in the development of autoimmune diseases, the impact of a single factor on the oxidative/antioxidant system in IBD is limited. Therefore, we have introduced a comprehensive index, the Oxidative Balance Score (OBS). The OBS is used to assess the overall exposure to pro-oxidants and antioxidants in an individual (11). A higher OBS indicates a more significant cumulative effect of antioxidant exposure. Recent studies have found an association between OBS and various diseases, such as non-alcoholic fatty liver (12) and metabolic syndrome (13). However, there is currently no research exploring the relationship between OBS and the occurrence of IBD and its related complications.

Considering the potential role of oxidative stress in IBD, the aim of this study is to investigate the association between OBS and the risk of developing IBD and its complications in a large follow-up study utilizing the UK BioBank, including patients with IBD, UC, and CD. Our findings will provide new evidence for the role of oxidative balance in IBD and its related complications. The study aims to deepen the understanding of the molecular mechanisms mediated by ROS and to further explore potential treatment strategies for IBD driven by oxidative stress.

## 2 Methods

### 2.1 Study population

**Figure 1:**
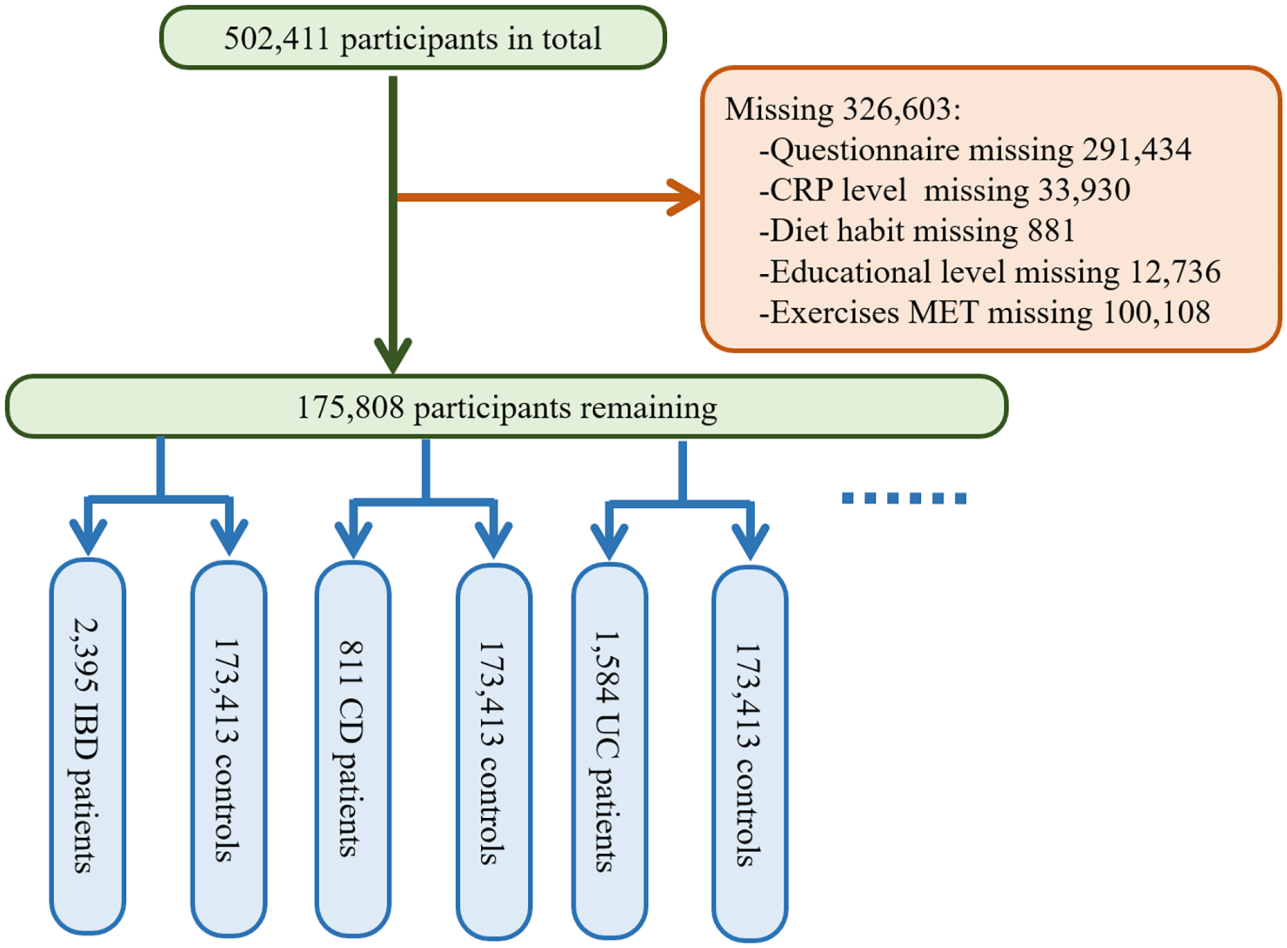
Sample Selection and Analysis Flowchart.

The UK BioBank database is the result of a large prospective cohort study that recruited more than 500,000 male and female participants aged 37 to 69 from various locations in the UK between 2006 and 2010 (14). The baseline data for the study were collected at this time, followed by four large-scale follow-ups and additional data collection. The database includes participants’ genetic information and blood samples, as well as data on lifestyle and environmental exposures, and tracks their health and medical records for several decades after. The National Health and Social Care Information Governance Board in England and Wales, as well as the Community Health Index Advisory Group in Scotland, approved the access to patient records for recruitment purposes. The UK BioBank obtained ethical approval from the Northwest Multi-center Research Ethics Committee. All participants signed informed consent using touchscreen signature devices. We excluded all the participants with incomplete questionnaire data (n=291,434) during recruitment, and further excluded participants with missing data on CRP levels (n=33,930), dietary habits (n=881), education level (n=12,736), and other covariates. In the end, a total of 175,808 participants were included in this study. Multiple methods were employed for handling missing data, such as mean imputation, multiple imputation, and exclusion. The exclusion criteria were established to ensure the quality and reliability of the study sample and to mitigate potential selection bias. More details are seen in **Figure 1**.

### 2.2 Inflammatory bowel disease

All diseases, medication prescriptions, and participant deaths were recorded in the database for utilization and management. Participants were followed up from recruitment until the diagnosis of crohn’s disease or ulcerative colitis, withdrawal from the study, date of death, or the last follow-up date, whichever came first. Assessment of inflammatory bowel disease was determined based on the International Classification of Diseases, 10th Revision (ICD-10) codes for primary hospital admission diagnoses (CD events were defined as “K50” and UC events were defined as “K51”).

### 2.3 Oxidative balance score

The calculation plan for OBS was based on prior empirical information (13, 15). This study included data on 5 lifestyle factors (smoking, alcohol consumption, tea consumption, body mass index, and physical activity) and 17 nutrient intake variables (total fat, iron, calcium, magnesium, vitamin B6, vitamin B12, vitamin C, vitamin D, vitamin E, total folate, carotenoids, dietary fiber, retinol, meat intake, vegetable intake, polyunsaturated fatty acids, saturated fatty acids), comprising 14 antioxidants and 8 prooxidants. The total OBS score was the sum of values (0, 1, 2) assigned based on given tertile groupings, with a value of 0 for lower tertiles and a value of 2 for higher tertiles, for both antioxidants and prooxidants. Ultimately, a score of 0 represented low levels of antioxidants, while a score of 2 indicated high levels. Thus, a higher OBS score indicated a more significant exposure to antioxidants. Smoking, alcohol consumption, and meat intake are handled differently to avoid issues of zero inflation in the scores. The scores for these variables are defined as 2 for never intake, and assigned values of 0 or 1 based on binarization according to the median. Vegetable intake was calculated by summing the intake of raw vegetables and cooked vegetables. Meat intake was calculated by summing the intake of beef, lamb, and pork. Physical activity was calculated by summing the weekly MET (Metabolic Equivalent Task) minutes of light, moderate, and vigorous physical activity. Due to the differential oxidative stress between genders, values were assigned separately for males and females based on prior empirical evidence (15). The data on these lifestyle factors and nutrient intake were obtained through a baseline completed Food frequency questionnaire (FFQ) by the participants. The questionnaire inquired about the intake frequency of fresh fruits, dried fruits, raw vegetables, cooked vegetables, oily fish, non-oily fish, processed meat, poultry, beef, lamb, pork, cheese, bread, cereal, tea, and drinking water. These intake frequencies were subsequently used to estimate the intake frequencies of essential nutrients such as energy, carbohydrates, and fats. MET data were derived based on the International Physical Activity Questionnaire (IPAQ) guidelines. For more specific details, please refer to **Table 1**.

**Table 1:** Components of the oxidative balance score.

OBS components	Unit	Property	Category	Female			Male		
				0	1	2	0	1	2
Total fat	g/day	P	Nutrient Intake	>82.15	56.19~82.15	<56.19	>94.27	64.54~94.27	<64.54
Iron	mg/day	P	Nutrient Intake	>14.59	10.77~14.59	<10.77	>16.26	12~16.26	<12
polyunsaturated fatty acids	g/day	P	Nutrient Intake	>15.5	8.89~15.5	<8.89	>17.59	10.08~17.59	<10.08
saturated fatty acids	g/day	P	Nutrient Intake	>31.32	20.66~31.32	<20.66	>36.36	23.91~36.36	<23.91
Smoking	package years	P	Lifestyle	>17	>0 & <17	=0	>21.5	>0 &<21.5	=0
Alcohol	g/day	P	Lifestyle	>19.52	>0 & <19.52	=0	>31.32	>0 &<31.32	=0
Meat	times/week	P	Lifestyle	>3	>0 & <3	=0	>3	>0 &<3	=0
BMI	kg/m2	P	Lifestyle	>28.30	24.34~28.30	<24.34	>29.00	25.79~29.00	<25.79
Carotene	ug/day	A	Nutrient Intake	<1370.22	1370.22~4147.08	>4147.08	<1014.02	1014.02~3374.59	>3374.59
Dietary fiber	g/day	A	Nutrient Intake	<12.78	12.78~18.49	>18.49	<12.99	12.99~19.13	>19.13
Vitamin B6	mg/day	A	Nutrient Intake	<1.72	1.72~2.37	>2.37	<1.87	1.87~2.58	>2.58
Total folate	ug/day	A	Nutrient Intake	<233.04	233.04~326.78	>326.78	<253.96	253.96~354.33	>354.33
Vitamin B12	mg/day	A	Nutrient Intake	<3.49	3.49~6.48	>6.48	<3.77	3.77~6.76	>6.76
Vitamin C	mg/day	A	Nutrient Intake	<91.45	91.45~178.59	>178.59	<82.29	82.29~168.05	>168.05
Vitamin E	mg/day	A	Nutrient Intake	<6.67	6.67~10.46	>10.46	<6.42	6.42~10.37	>10.37
Calcium	mg/day	A	Nutrient Intake	<749.84	749.84~1049.64	>1049.64	<803.12	803.12~1122.7	>1122.7
Magnesium	mg/day	A	Nutrient Intake	<280.59	280.59~365.89	>365.89	<308.35	308.35~404.4	>404.4
Retinol	ug/day	A	Nutrient Intake	<198.99	198.99~359.63	>359.63	<222.51	222.51~407.21	>407.21
Vitamin D	ug/day	A	Nutrient Intake	<1.03	1.03~2.45	>2.45	<1.2	1.2~2.76	>2.76
Physical activity	MET minutes/week	A	Lifestyle	<1062	1062~2685.17	>2685.17	<1108.5	1108.5~2897.17	>2897.17
Tea	cups/day	A	Lifestyle	<2	2~4	>4	<2	2~4	>4
vegetable	tablespoons/day	A	Lifestyle	<4	4~6	>6	<3	3~5	>5

OBS, Oxidative Balance Score; A, antioxidant; P, pro-oxidant; MET, metabolic equivalent of task; BMI, body mass index.

### 2.4 Covariate assessment

In the current study, the following variables were considered as covariates: age, race (European ancestry, Asian ancestry excluding Chinese, African ancestry, Chinese ancestry, other ethnicities, mixed ancestry), educational performance, and Townsend deprivation index. Ethnic background may influence individual genetics, environmental exposures, and lifestyle choices, thereby impacting the occurrence of IBD. Educational level typically reflects an individual’s cognitive abilities and social resources, which may be related to the oxidative balance score and the risk of IBD onset investigated in our study. Additionally, to account for individual differences in overall intake and spontaneous oxidative stress, we also included dietary energy intake and high-sensitivity CRP as covariates. Age, race and education level information were assessed through self-reported questionnaires. The acquisition of educational attainment indicators was divided into two subdomains: one related to children and adolescents, and one related to adult skills. These two subdomains aimed to reflect the “flow” and “stock” of educational disadvantage within a region, respectively. Dietary energy intake was calculated using data obtained from the aforementioned dietary questionnaire. The serum high-sensitivity CRP data were obtained through highly sensitive immunoassay testing conducted by skilled researchers using the Beckman Coulter AU5800 system.

### 2.5 Statistical analysis

Continuous variables were expressed as mean (± standard deviation) in baseline descriptions, and differences were described using the Krusal-Wallis test. Categorical variables were described as sample counts (percentages), and statistical differences were described using Pearson’s Chi-squared test. In this study, we categorized the continuous OBS scores based on quartiles (Q1: <25th percentile, Q2: 25-50th percentile, Q3: 50-75th percentile, Q4: 75-100th percentile). We used Cox proportional hazards model to explore the association between OBS and the occurrence of IBD, including its two subtypes (UC and CD). The baseline of this study was set at the time when participants completed the dietary questionnaire, with the censoring date being June 1, 2022, and the date of questionnaire obtained from field number “105010”. The follow-up period was calculated by subtracting the date of the questionnaire from either the date of onset or the censor date, varying for each participant. The distribution of follow-up times for all participants is shown in **Supplementary Figure 1**. In this study, three models were used to investigate the association between OBS and IBD, UC, and CD. Model 1 was adjusted for age, race, educational attainment indicators, and the Townsend Deprivation Index. These basic variables help control for confounding effects of fundamental socioeconomic and demographic characteristics. Model 2 further adjusted for dietary energy intake. This adjustment considers the direct impact that dietary factors might have on the development of OBS and IBD, allowing for a more accurate assessment of the pure relationship between OBS and IBD by eliminating the interference of dietary habits. Model 3 additionally adjusted for serum CRP level based on Model 2. As an inflammatory marker, adjusting for CRP helps evaluate the role of chronic inflammation in the relationship between OBS and IBD, thereby providing insights into how inflammation-mediated physiological mechanisms might influence the association between these two conditions. Furthermore, we conducted subgroup analyses based on gender and age groups to further investigate the impact of OBS on IBD, UC, and CD in different gender. Additionally, using the aforementioned three models, we further investigated the correlation between OBS scores and complications associated with IBD (abscess, obstruction, perforation, fistula, megacolon, Clostridium difficile infection, sepsis). Subgroup analyses of OBS and IBD-related complications were conducted among different gender. Finally, sensitivity analyses were performed by reanalyzing the data based on different types of OBS contributions and using the leave-one-out method to examine the impact of removing a specific score contribution.

### 2.6 Data acquisition and statistical software

The UK Biobank data were obtained through our application with ID 84347. Data were queried and downloaded using the web tool at https://biobank.ndph.ox.ac.uk/ukb/. Fields of interest were queried in the “Search” section. Specific data downloading and decompression software were downloaded from the “Download” section. Subsequently, fields were downloaded based on their respective field ID. The data were then imported into R (version 4.2.3) for data cleaning and analysis.

## 3 Results

### 3.1 Baseline characteristics

The baseline characteristics of the participants stratified into quartiles of OBS are presented in **Table 2**. Females accounted for a higher proportion (53%), and the mean ages of the subjects was 55.9 ± 8.0 years. Compared with participants in the lowest quartile of OBS, those in the highest quartile were more likely to be (1) females, (2) of European descent, (3) older, (4) have higher dietary energy intake, and (5) have lower Townsend Deprivation Index, BMI, educational attainment indicators, and CRP levels. They also had lower rates of emergency hospitalization and mortality. There is no statistically significant difference in the rate of IBD-related surgeries among OBS groups. A total of 2,395 IBD patients were included in this study. Of these, there were 811 CD patients and 1,584 UC patients.

**Table 2:** Baseline characteristics of participants according to quartiles of oxidative balance score.

	Overall N = 175,808	Quantile OBS groups				
		Q1 [4,19) N = 40,379	Q2 [19,22) N = 35,004	Q3 [22,26) N = 49,209	Q4 [26,41) N = 51,216	p-value
OBS score, Mean (SD)	22.5 (5.0)	15.8 (2.1)	20.0 (0.8)	23.5 (1.1)	28.5 (2.2)	<0.001
IBD, n (%)	2,395 (1.4%)	602 (1.5%)	491 (1.4%)	659 (1.3%)	643 (1.3%)	0.019
CD, n (%)	811 (0.5%)	214 (0.5%)	164 (0.5%)	247 (0.5%)	186 (0.4%)	<0.001
UC, n (%)	1,584 (0.9%)	388 (1.0%)	327 (0.9%)	412 (0.8%)	457 (0.9%)	0.2
Gender, n (%)						<0.001
Female	94,044 (53%)	20,971 (52%)	18,392 (53%)	26,215 (53%)	28,466 (56%)	
Male	81,764 (47%)	19,408 (48%)	16,612 (47%)	22,994 (47%)	22,750 (44%)	
TDI score, Mean (SD)	-1.6 (2.9)	-1.3 (3.0)	-1.6 (2.9)	-1.7 (2.8)	-1.8 (2.8)	<0.001
missing	221	68	39	63	51	
Education score, Mean (SD)	11.7 (13.8)	13.3 (15.1)	11.7 (13.8)	11.2 (13.2)	11.0 (13.0)	<0.001
missing	4,482	1,108	871	1,199	1,304	
Ethnicity, n (%)						<0.001
European	167,952 (96%)	38,319 (95%)	33,409 (96%)	47,162 (96%)	49,062 (96%)	
Mixed-race	1,054 (0.6%)	299 (0.7%)	197 (0.6%)	282 (0.6%)	276 (0.5%)	
Asian	2,488 (1.4%)	583 (1.4%)	526 (1.5%)	699 (1.4%)	680 (1.3%)	
African	2,082 (1.2%)	706 (1.8%)	410 (1.2%)	467 (1.0%)	499 (1.0%)	
Chinese	495 (0.3%)	90 (0.2%)	106 (0.3%)	142 (0.3%)	157 (0.3%)	
Others	1,239 (0.7%)	268 (0.7%)	253 (0.7%)	333 (0.7%)	385 (0.8%)	
Missing	498	114	103	124	157	
BMI, Mean (SD), Kg/m^2^	26.9 (4.6)	28.3 (4.9)	27.1 (4.6)	26.6 (4.4)	25.8 (4.2)	<0.001
Age, Mean (SD)	55.9 (8.0)	55.0 (8.0)	55.7 (8.0)	56.0 (7.9)	56.6 (7.9)	<0.001
CRP level, Mean (SD), mg/L	2.3 (4.0)	2.7 (4.3)	2.3 (3.9)	2.2 (4.0)	1.9 (3.7)	<0.001
missing	10,192	2,377	2,009	2,868	2,938	
Daily energy intake, Mean (SD), KJ	8,862.8 (3,036.6)	7,731.7 (2,481.1)	8,300.7 (2,718.7)	9,135.5 (2,997.0)	9,876.7 (3,285.3)	<0.001
Emergency admission, n (%)	83,369 (47%)	20,236 (50%)	16,449 (47%)	22,933 (47%)	23,751 (46%)	<0.001
IBD-related operation, n (%)	376 (0.2%)	100 (0.2%)	68 (0.2%)	99 (0.2%)	109 (0.2%)	0.4
Death, n (%)	8,670 (4.9%)	2,337 (5.8%)	1,670 (4.8%)	2,283 (4.6%)	2,380 (4.6%)	<0.001

OBS, Oxidative Balance Score; TDI, Thomson Deprivation Index;CD, Crohn’s disease; UC, Ulcerative colitis; IBD, inflammatory bowel disease; SD, standard deviation; BMI, body mass index; CRP, C-reactive protein; Education refered to the age of Highest Level of Education

### 3.2 Association between OBS and inflammatory bowel disease and its subtypes

According to the Cox proportional hazards model, OBS was found to be associated with the occurrence of IBD. In model 1, for every one-point increase in the continuous OBS score, the incidence of IBD decreases by 1.0% (HR 0.990, 95%CI 0.982-0.998, p=0.016). Compared to the lowest quartile of OBS (Q1 group), the risk of developing IBD was reduced by 15.0% in the highest quartile (Q4 group) (HR 0.850, 95%CI 0.758-0.952, p=0.005). In model 2, for every one-point increase in the continuous OBS score, the incidence of IBD decreases by 1.5% (HR 0.985, 95%CI 0.977-0.993, p<0.001). Compared to the Q1 group, the risk of developing IBD was reduced by 13.0% in the Q3 group (HR 0.870, 95%CI 0.776-0.975, p=0.017) and 21.1% in the Q4 group (HR 0.789, 95%CI 0.702-0.888, p<0.001). In model 3, for every one-point increase in the continuous OBS score, the incidence of IBD decreases by 1.2% (HR 0.988, 95%CI 0.980-0.997, p=0.009). Compared to the Q1 group, the risk of developing IBD was reduced by 17.4% in the Q4 group (HR 0.826, 95%CI 0.732-0.933, p=0.002).

OBS is associated with the occurrence of UC. In model 1, no significant correlation was found between continuous and four-category OBS and UC (All P-values > 0.05). However, in model 2, compared to the Q1 group, the risk of developing UC was reduced by 17.3% in the Q3 group (HR 0.827, 95%CI 0.716-0.954, p=0.009) and 14.6% in the Q4 group (HR 0.850, 95%CI 0.740-0.986, p=0.031). In model 3, compared to the Q1 group, the risk of developing UC was reduced by 17.1% in the Q3 group (HR 0.829, 95%CI 0.715-0.960, p=0.012) and 14.1% in the Q4 group (HR 0.859, 95%CI 0.741-0.995, p=0.043).

**Figure 2:**
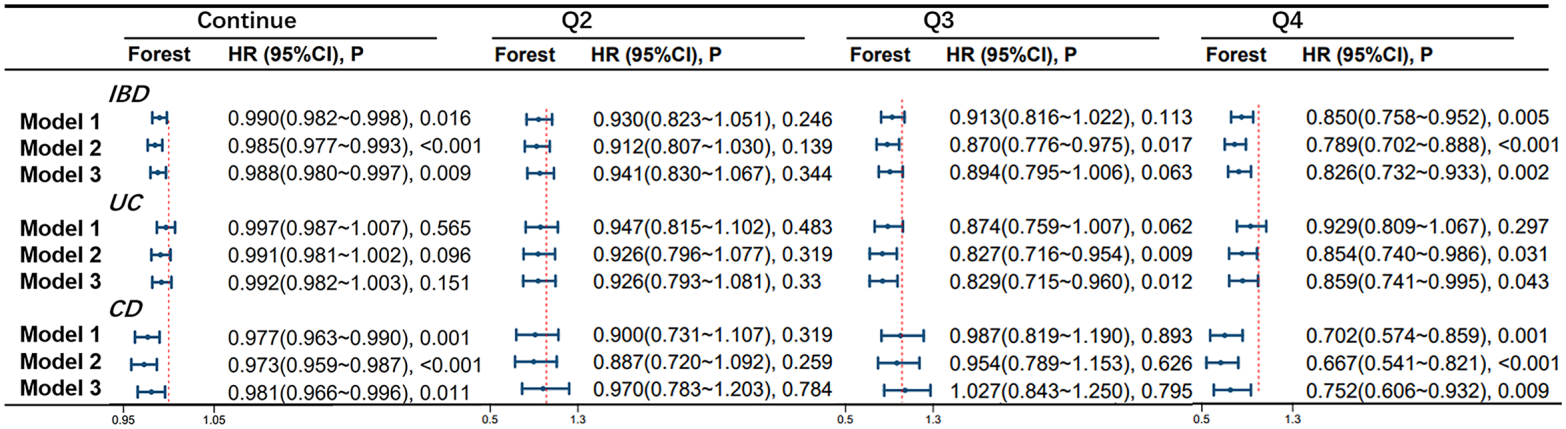
Association of Oxidative Balance Score with Inflammatory Bowel Disease and Subtypes. The hazard ratio value of Q2-Q4 is based on the Q1 group as the reference. Model 1 was adjusted for age, race, educational performance indicators, and Townsend deprivation index. Model 2, building upon Model 1, was further adjusted for dietary energy intake. Model 3, extending the adjustments made in Model 2, additionally accounted for plasma CRP concentration.IBD, inflammatory bowel disease; UC, ulcerative colitis; CD, Crohn’s disease; HR, hazard ratio.

OBS is associated with the occurrence of CD. In model 1, for every one-point increase in the continuous OBS score, the incidence of CD decreases by 2.3% (HR 0.977, 95%CI 0.963-0.990, p=0.001). Compared to the Q1 group, the risk of developing CD was reduced by 29.8% in the Q4 group (HR 0.702, 95%CI 0.574-0.859, p=0.001). In model 2, for every one-point increase in the continuous OBS score, the incidence of CD decreases by 2.7% (HR 0.973, 95%CI 0.959-0.987, p<0.001). Compared to the Q1 group, the risk of developing CD was reduced by 33.3% in the Q4 group (HR 0.667, 95%CI 0.541-0.821, p<0.001). In model 3, for every one-point increase in the continuous OBS score, the incidence of CD decreases by 2.9% (HR 0.981, 95%CI 0.966-0.996, p=0.011). Compared to the Q1 group, the risk of developing CD was reduced by 24.8% in the Q4 group (HR 0.752, 95%CI 0.606-0.932, p=0.009). More specific details can be seen in **Figure 2**.

### 3.3 Association between OBS and UC and CD stratified by gender

OBS is significantly associated with the occurrence of UC in the female population. In model 1, compared to the Q1 group, the risk of developing UC was reduced by 26.3% in the Q2 group (HR 0.737, 95%CI 0.587-0.925, p=0.009), 19.9% in the Q3 group (HR 0.801, 95%CI 0.654-0.981, p=0.032), and 19.4% in the Q4 group (HR 0.806, 95%CI 0.661-0.983, p=0.034) in the female population. In model 2, compared to the Q1 group, the risk of developing UC was reduced by 27.4% in the Q2 group (HR 0.726, 95%CI 0.578-0.912, p=0.006), 23.1% in the Q3 group (HR 0.769, 95%CI 0.626-0.944, p=0.012), and 24.2% in the Q4 group (HR 0.758, 95%CI 0.618-0.931, p=0.008) in the female population. In model 3, compared to the Q1 group, the risk of developing UC was reduced by 25.3% in the Q2 group (HR 0.747, 95%CI 0.592-0.944, p=0.015), 21.4% in the Q3 group (HR 0.786, 95%CI 0.636-0.971, p=0.025), and 22.5% in the Q4 group (HR 0.775, 95%CI 0.627-0.958, p=0.019) in the female population.

**Figure 3:**
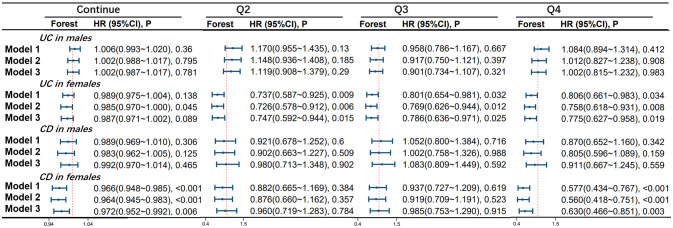
Association of Oxidative Balance Score with Stratified Age Groups in UC and CD. The hazard ratio value of Q2-Q4 is based on the Q1 group as the reference. Model 1 was adjusted for age, race, educational performance indicators, and Townsend deprivation index. Model 2, building upon Model 1, was further adjusted for dietary energy intake. Model 3, extending the adjustments made in Model 2, additionally accounted for plasma CRP concentration.IBD, inflammatory bowel disease; UC, ulcerative colitis; CD, Crohn’s disease; HR, hazard ratio; CI, confidence interval.

There is a significant correlation between OBS and CD occurrence in the female population. In model 1, for every one-point increase in the continuous OBS score, the incidence of CD decreases by 3.4% (HR 0.966, 95%CI 0.948-0.985, p<0.001). Compared to the Q1 group, the risk of CD occurrence was reduced by 42.3% in the Q4 group (HR 0.577, 95%CI 0.434-0.767, p<0.001). In model 2, for every one-point increase in the continuous OBS score, the incidence of CD decreases by 3.6% (HR 0.964, 95%CI 0.945-0.983, p<0.001). Compared to the Q1 group, the risk of CD occurrence was reduced by 44.0% in the Q4 group (HR 0.560, 95%CI 0.418-0.751, p<0.001). In model 3, for every one-point increase in the continuous OBS score, the incidence of CD decreases by 2.8% (HR 0.972, 95%CI 0.952-0.992, p=0.006). Compared to the Q1 group, the risk of CD occurrence was reduced by 37.0% in the Q4 group (HR 0.630, 95%CI 0.466-0.851, p=0.003). However, no significant correlation between OBS and CD was observed in the male population (see **Figure 3**).

### 3.4 Differences of dietary balance scores in age-stratified female CD patients

**Figure 4:**
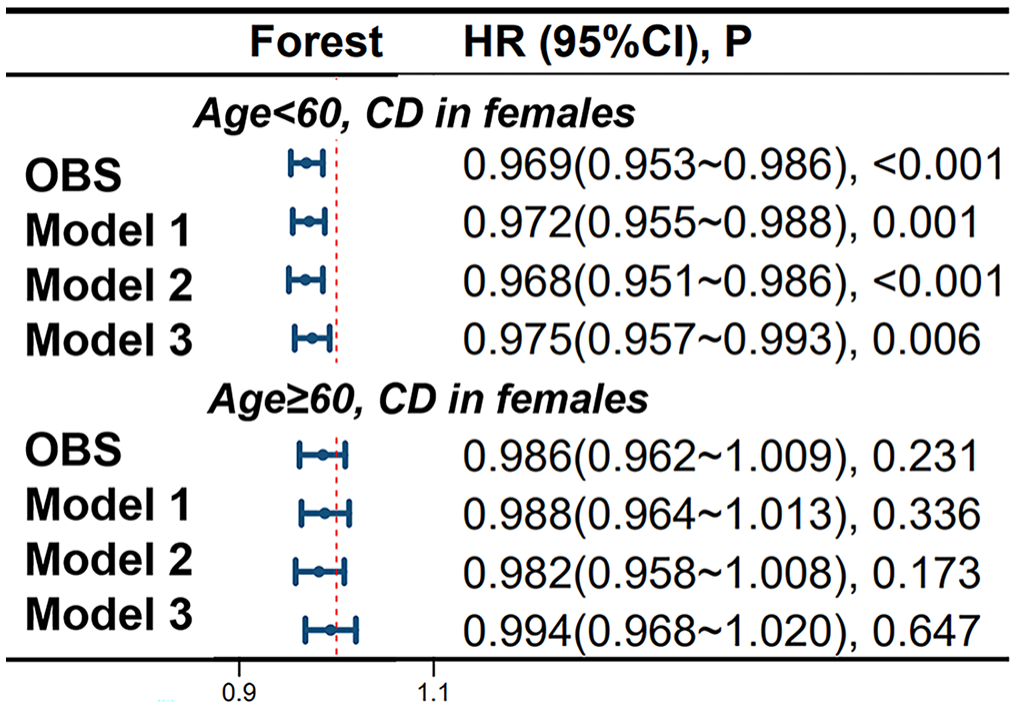
Differential Impact of Oxidative Balance Score in Age-Stratified Female CD Patients. Model 1 was adjusted for age, race, educational performance indicators, and Townsend deprivation index. Model 2, building upon Model 1, was further adjusted for dietary energy intake. Model 3, extending the adjustments made in Model 2, additionally accounted for plasma CRP concentration. CD, Crohn’s disease; HR, hazard ratio; CI, confidence interval; OBS, Oxidative Balance Score.

In female CD patients, using 60 years as a cutoff point, in the age < 60 group, continuous OBS is significantly negatively correlated with CD occurrence in models 1, 2, and 3, with adjusted HRs of 0.972 (95%CI 0.955-0.988), 0.968 (95%CI 0.951-0.986) and 0.975 (95%CI 0.957-0.993), respectively. However, in the age > 60 group of female patients, no significant correlation was found between continuous OBS and the occurrence of CD. More specific details can be seen in **Figure 4**.

### 3.5 Correlation of dietary balance score with intestinal obstruction complications in CD patients

**Figure 5:**
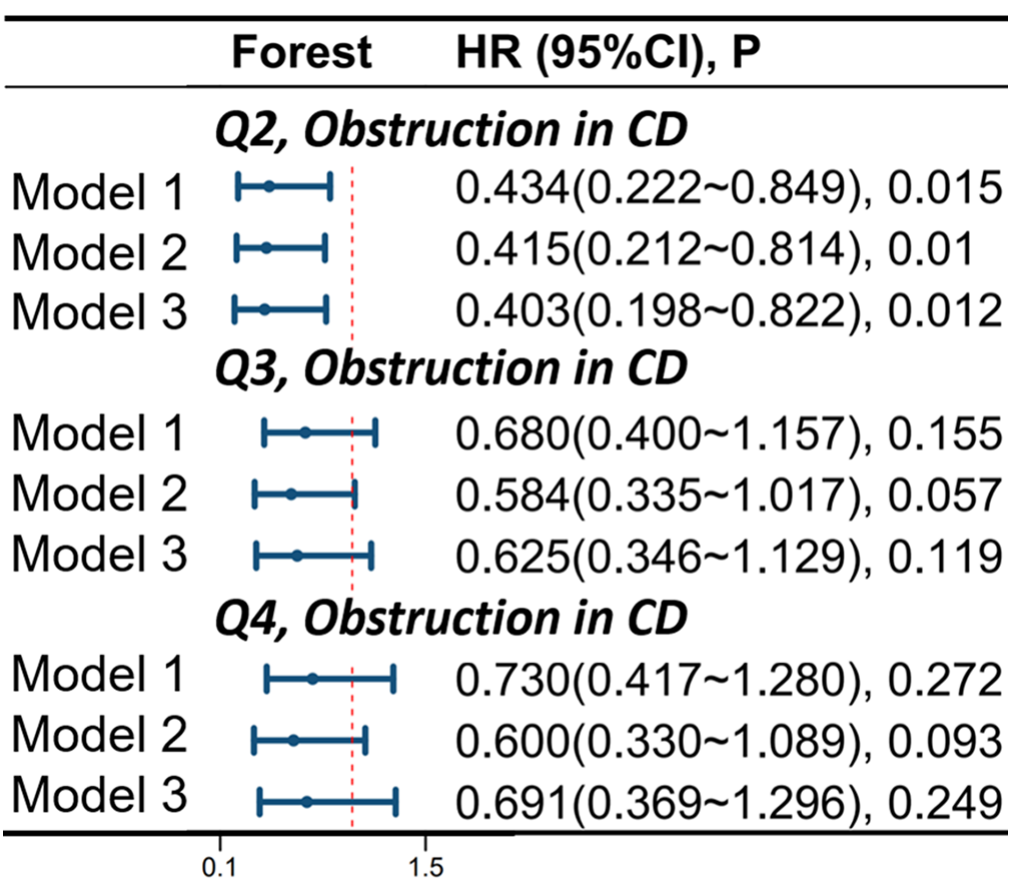
Correlation of Oxidative Balance Score with Complications of Intestinal Obstruction in CD Patients. The hazard ratio value of Q2-Q4 is based on the Q1 group as the reference. Model 1 was adjusted for age, race, educational performance indicators, and Townsend deprivation index. Model 2, building upon Model 1, was further adjusted for dietary energy intake. Model 3, extending the adjustments made in Model 2, additionally accounted for plasma CRP concentration. CD, Crohn’s disease; HR, hazard ratio; CI, confidence interval.

There is a correlation between OBS and intestinal obstruction in CD patients. In model 1, compared to the Q1 group, the risk of obstruction occurrence was reduced by 56.6% in the Q2 group (HR 0.434, 95%CI 0.222-0.849, p=0.015). In model 2, compared to the Q1 group, the risk of obstruction occurrence was reduced by 58.5% in the Q2 group (HR 0.415, 95%CI 0.212-0.814, p=0.010). In model 3, compared to the Q1 group, the risk of obstruction occurrence was reduced by 59.7% in the Q2 group (HR 0.403, 95%CI 0.198-0.822, p=0.012). For more details, please refer to **Figure 5**.

### 3.6 Sensitivity analysis

**Figure 6:**
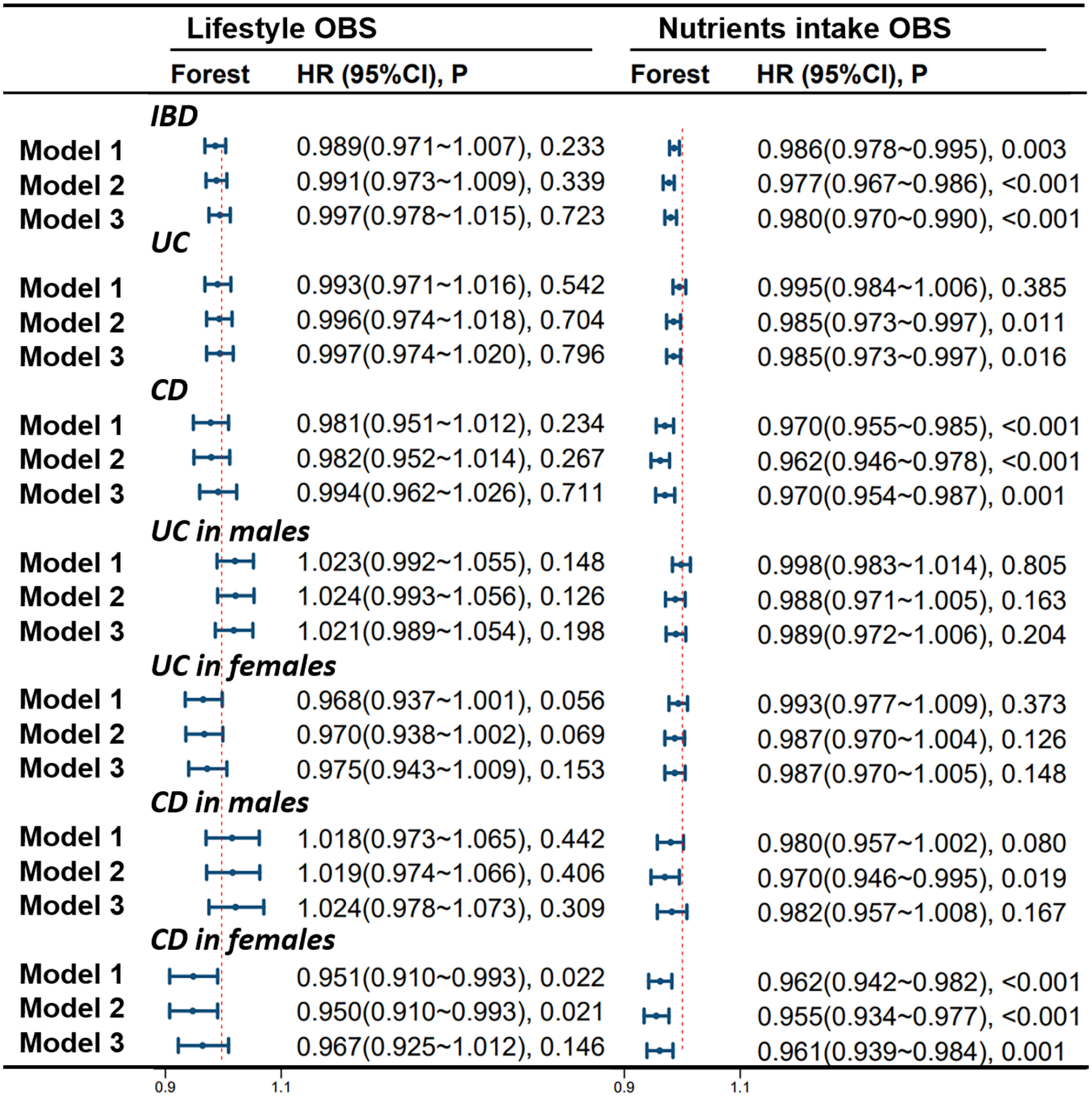
Stratified Study on the Contribution of Lifestyle and Dietary Intake to OBS. Model 1 was adjusted for age, race, educational performance indicators, and Townsend deprivation index. Model 2, building upon Model 1, was further adjusted for dietary energy intake. Model 3, extending the adjustments made in Model 2, additionally accounted for plasma CRP concentration. IBD, inflammatory bowel disease; UC, ulcerative colitis; CD, Crohn’s disease; HR, hazard ratio; CI, confidence interval; OBS, Oxidative Balance Score.

**Figure 7:**
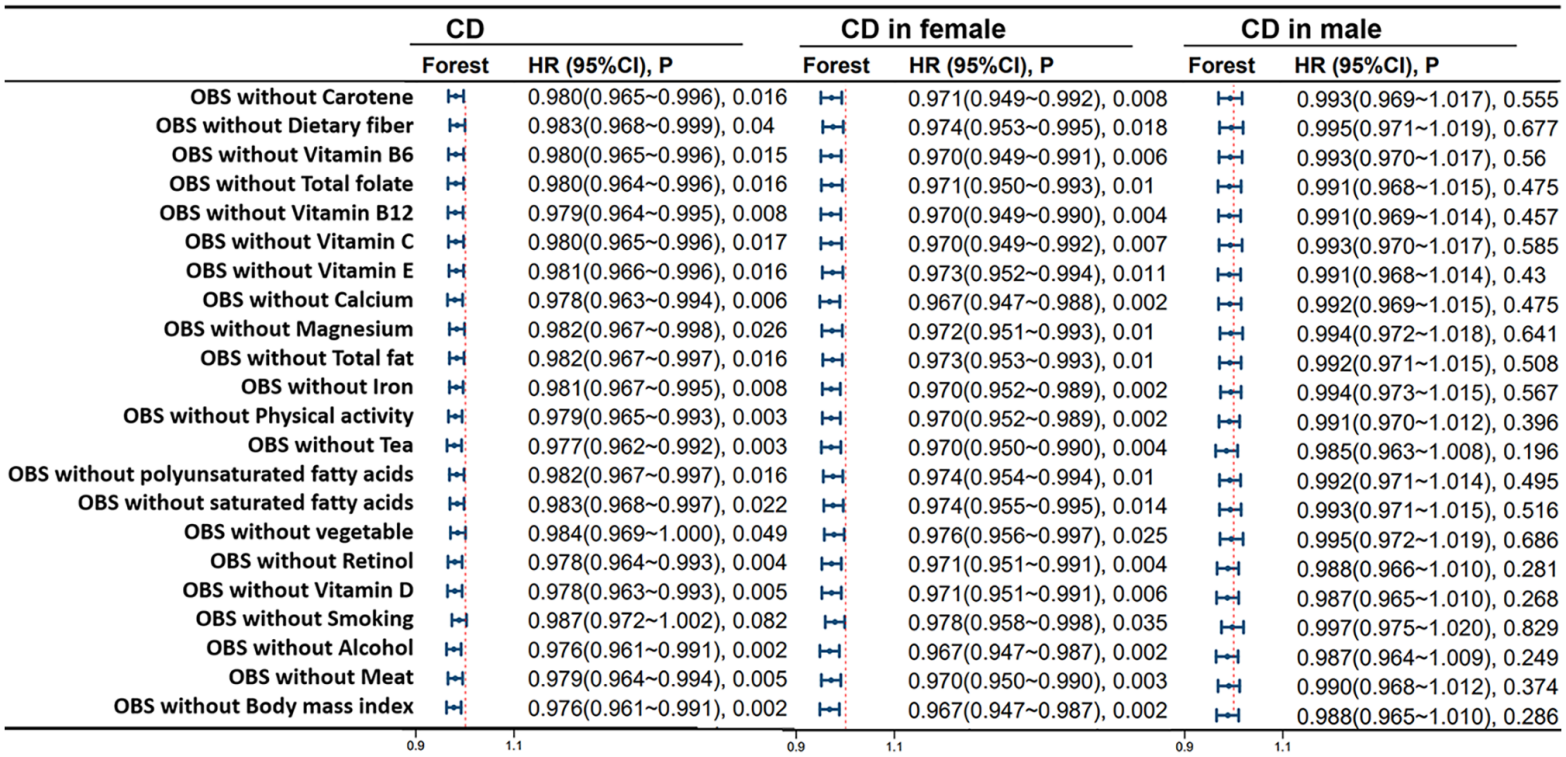
Leave-One-Out Analysis for OBS in CD Patients. Model 3 was adjusted for age, race, educational performance indicators, Townsend deprivation index, dietary energy intake and plasma CRP concentration. CD, Crohn’s disease; HR, hazard ratio; CI, confidence interval; OBS, Oxidative Balance Score.

**Figure 6** shows the correlation of lifestyle OBS and dietary OBS with IBD, UC, and CD in the overall participants and different gender subgroups. There is a significant and stable negative correlation between dietary OBS and the occurrence of IBD, UC, and CD in patients. In gender-stratified subgroup analysis, both diet-related OBS and lifestyle-related OBS are negatively correlated with the occurrence of CD in females. In addition, we also conducted a leave-one-out analysis, which means re-analyzing the data after removing the contribution of a certain component score of OBS, and concluded that there is still a significant negative correlation between OBS and CD occurrence in CD patients and female participants. However, no significant correlation between OBS and CD occurrence was observed in the male population. For more details, please refer to **Figure 7**.

## 4 Discussion

In recent years, a growing body of epidemiological evidence has suggested that oxidative stress may play a major role in the pathogenesis of IBD (3). In this large-scale prospective study involving adult participants in the United Kingdom, we observed a negative correlation between overall OBS and dietary OBS with the incidence of IBD, UC, and CD, with a more pronounced effect in female participants. Additionally, we found a negative correlation between higher OBS and the occurrence of intestinal obstruction in CD patients. These findings remained robust even after adjusting for various potential confounders and conducting additional analyses stratified by gender and subgroups.

To the best of our knowledge, this is the first study to investigate the association between OBS and inflammatory bowel disease. While previous studies have shown that substances such as vitamin D and reactive oxygen species can reduce the risk and severity of IBD by alleviating oxidative stress, most of these studies have only used one or a few circulating biomarkers reflecting oxidative stress, such as 8-OHdG and isoprostanes (16, 17). Some studies on chronic diseases have suggested that the combination of multiple factors may have a stronger association with disease risk than considering individual nutrients alone (18, 19). This study, on the other hand, thoroughly assessed the oxidative stress-related exposure by calculating the overall intake of various pro-oxidants and antioxidants using the OBS derived from dietary patterns and lifestyle choices, providing a comprehensive assessment of the overall oxidative balance in the body. Moreover, our study extensively investigated the association between OBS and the risk and severity of inflammatory bowel disease and its complications through long-term follow-up, providing a solid foundation for further optimization of treatment and care strategies for IBD patients. In this study, differences in baseline characteristics that across quartiles of OBS reflect variations in physiological and biochemical statuses among patient groups with different OBS quartiles. By analyzing inter-group differences, OBS may be closely associated with physiological processes such as inflammatory status and immune function. The group with higher OBS quartiles may exhibit elevated levels of oxidative stress and inflammatory markers, suggesting a potential correlation between oxidative stress and the onset and progression of IBD.

Oxidative stress plays a crucial and multifaceted role in the complex pathogenesis of inflammatory bowel disease. The production of ROS in the gastrointestinal tract may lead to further oxidative stress damage to intestinal cells. The gastrointestinal tract is one of the main sources of ROS in the body, and the intake of substances and intestinal pathogens can promote inflammation, leading to oxidative stress (OS) (20, 21). Intestinal epithelial cells, along with inflammatory cells, participate in the development of inflammation by producing ROS/RNS (reactive nitrogen species) under inflammatory conditions. One of the characteristics of the pathogenesis of IBD is the imbalance between helper T cells and regulatory T cells. The excessive production of Th1-cell-mediated inflammatory cytokines such as IL-12, IL-17, and IL-23 leads to the development of CD (22, 23). These pro-inflammatory cytokines, in combination with other inflammatory cells, promote the production of ROS (20). These ROS, mainly including superoxide and hydroxyl radicals, together with pro-inflammatory cytokines, cause cellular damage and tissue destruction (24). In the intestinal mucosa, ROS can disrupt tight junctions between cells, increase intestinal permeability, ultimately leading to the disruption of the intestinal epithelial barrier and further inflammation (25). Moreover, although low to moderate levels of ROS have a role in resisting pathogen invasion and promoting tissue repair, excessive ROS produced during intense inflammatory responses in IBD can have detrimental effects on the intestinal mucosa. Therefore, based on the above mechanisms, it is evident that ROS are involved in the pathogenesis of IBD. However, antioxidants and compounds can mitigate the multifaceted effects of oxidative stress through various mechanisms. For example, both enzymatic and non-enzymatic antioxidants have a balancing effect on oxidative stress (4). These antioxidants directly form an antioxidant network (26), collectively combating lipid, protein, and DNA damage caused by ROS (9). Furthermore, various antioxidants such as vitamin C, vitamin E, carotenoids, etc., can neutralize ROS, preventing downstream oxidative reactions, aiding in the prevention of damage and shedding of intestinal epithelial cells, and maintaining the integrity of intestinal mucosal epithelium (27). Additionally, substances such as butyrate can reduce oxidative stress in colonic epithelial cells by inhibiting the activity of the NF-kB pathway, alleviating intestinal inflammation and fibrotic reactions, preserving the vitality of intestinal tissues, thereby reducing the risk of intestinal obstruction (28).

Our results also showed a gender dimorphism in the effect of OBS on the development of IBD. In female patients, particularly in the >60 age group, OBS scores significantly reduced the risk of CD incidence. The decline in estrogen levels during menopause may contribute to this outcome. In female patients, higher OBS was associated with a reduced risk of IBD, UC, and CD, with a more pronounced effect in CD patients. Hormonal differences may be responsible for these findings. In a cross-sectional study by Rolston et al. (29), it was reported that female patients with inflammatory bowel disease experienced worsening of symptoms during the menstrual cycle, with a more pronounced effect in CD patients compared to UC patients. The antioxidant effects of estrogen may be mediated through increased expression of antioxidant enzymes (AOEs) (30, 31). Changes in estrogen levels can also affect the expression of inflammatory factors (4). Estrogen can also maintain intestinal epithelial barrier function by affecting epithelial and mucus formation, epithelial permeability, and chloride ion secretion (32). Interestingly, activation of G-protein coupled estrogen receptor (GPER) can reduce mortality in animal models of CD, improve macroscopic and microscopic scores, and decrease CRP levels, which is achieved through the regulation of extracellular signal-regulated kinase (ERK) signaling pathway, involvement in signal transduction and immune response gene expression, and modulation of certain RNA expression (33). In addition, the A-ring phenolic hydroxyl on estrogen is a determining factor in free radical clearance and possesses antioxidant properties independent of activation of estrogen receptors and hormone function (34). Furthermore, sensitivity analysis suggests that both dietary OBS and lifestyle OBS significantly reduce the risk of CD incidence in females, possibly indicating a close association with gender-specific differences in dietary behaviors. Women tend to be more concerned about managing their physique and are prone to engaging in behaviors associated with eating disorders, such as dieting. This may result in inadequate nutritional intake for women, increasing their risk of various types of diseases compared to men (35). Moreover, the absence of a correlation between OBS and IBD among males may not only be attributed to the differences in hormone levels and dietary habits between genders but could also be closely linked to variations in gene expression that influence inflammation and oxidative stress. Research indicates that females are more likely to demonstrate genetic susceptibility to X-chromosome-associated IBD compared to males (36). Our findings provide additional evidence for the gender-specific etiology of IBD and highlight the different effects of OBS on the development of IBD according to gender, thus reinforcing the relevance of OBS in the assessment of IBD susceptibility.

There are several obvious strengths in this study. First, this study provides preliminary evidence that higher antioxidant exposure assessed by OBS is associated with reduced risk of IBD and its complications. Our study is based on OBS, a comprehensive measure of oxidative balance, rather than single biomarkers, to capture the complex relationships between various factors and to investigate the related outcomes comprehensively. Second, the participant data in this study are from a large database, the UK Biobank, which has a large sample size and can effectively avoid sampling bias. Third, this study controlled for many confounding factors, including demographic characteristics, diet quality, dietary energy, blood biomarkers, etc., to maximize the internal validity of the study. Fourth, this study conducted stratified analysis by gender and found that OBS has a more protective effect on female IBD patients, especially those with CD, making the results more targeted. Therefore, these findings have additional public health implications for better managing antioxidant-rich diets and lifestyles to more effectively prevent CD. However, this study also has several limitations. First, due to limitations of the database, the number of cases for some IBD complications is less than 30, which may introduce bias in the conclusions by not meeting the assumptions of regression analysis. Second, under the assumption that all pro-oxidants and antioxidants are linearly related to oxidative stress, this study overlooks the threshold effects of antioxidants (37, 38). However, it has been demonstrated that certain antioxidants may exhibit pro-oxidative activity at high doses or under certain conditions, thus the association of specific antioxidants may be uncertain. Third, this study was unable to accurately measure and collect the long-term dynamic changes of OBS components, including lifestyle components, and the observational design of the study limits causal inferences to some extent.

Despite these limitations, our study is the first to report a significant association between OBS and IBD in a large, population-based prospective study with long-term follow-up. This provides clinicians with an accurate and feasible risk prediction factor which can be used for assessing individual disease risk, especially for those at risk of IBD, as an intervention measure. This indicator can also be used for the development of screening and preventive strategies. Additionally, in view of the gender differences in the impact of OBS on female IBD patients, clinicians should pay more attention to the treatment and care of female IBD patients. Understanding the influence of estrogen on antioxidant activity can assist doctors in better managing the disease in female IBD patients. Finally, a comprehensive treatment strategy, including controlling pro-oxidants and increasing antioxidant intake, may be helpful in improving treatment outcomes for IBD patients. Clinicians should consider exploring the optimization of antioxidant status through diet, supplements, and lifestyle to improve the prognosis and prevention of IBD, thereby bringing more significant clinical benefits to IBD patients.

## 5 Conclusion

We found a significant negative correlation between OBS and IBD, particularly in female CD patients. This study emphasizes the crucial role of antioxidant diet and lifestyle, potentially conferring greater advantages for female CD patients.

## Data availability statement

The raw data supporting the conclusions of this article will be made available by the authors, without undue reservation.

## Ethics statement

The studies involving humans were approved by The North West Multi-center Research Ethics Committee, Manchester, U.K. (REC reference for UK Biobank 11/NW/0382). The studies were conducted in accordance with the local legislation and institutional requirements. The participants provided their written informed consent to participate in this study.

## Author contributions

FL: Writing – original draft, Visualization, Project administration, Formal analysis, Data curation. YC: Writing – original draft, Software, Methodology, Investigation, Data curation. ZhaW: Writing – original draft, Validation, Investigation. ZhiW: Writing – original draft, Validation, Investigation. QZ: Writing – original draft, Validation, Investigation. XH: Writing – original draft, Validation, Investigation. ZX: Writing – original draft, Validation, Investigation. CY: Writing – original draft, Investigation. YueL: Writing – original draft, Investigation. SC: Writing – original draft, Investigation. HL: Writing – original draft, Investigation. SH: Writing – original draft, Investigation. YuqL: Writing – review & editing, Supervision, Resources, Conceptualization. TT: Writing – review & editing, Supervision, Resources.
