# Supplementary material for: Antioxidant insights: investigating the protective role of oxidative balance in inflammatory bowel disease
Source: Front Endocrinol (Lausanne). 2024 May 31;15:1386142. doi: 10.3389/fendo.2024.1386142 (PMC11176441; doi:10.3389/fendo.2024.1386142)

**Supplementary Figure 1: Histogram of Follow-up Duration (Years) for Participants in This Study**

*Note: Follow-up duration = (Date of follow-up completion - Date of questionnaire completion)÷ 365.25.*


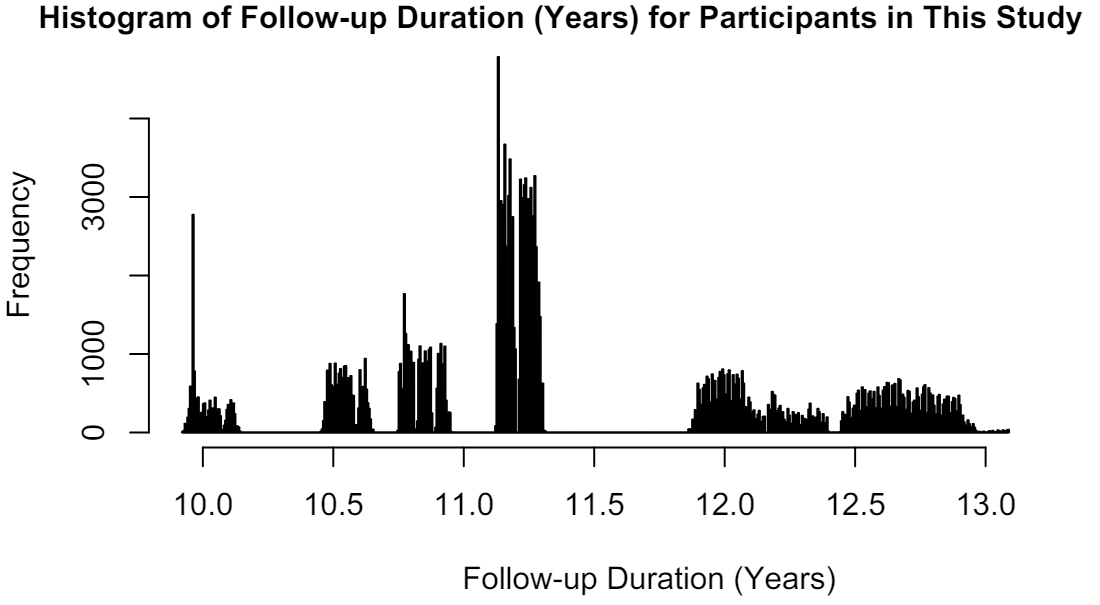

Supplement: Supplementary file 1 [file DataSheet_1.docx]
